# Implementation of an educational intervention among Iranian hajj pilgrims for the prevention of influenza-like illness

**DOI:** 10.1186/2047-2994-4-S1-O48

**Published:** 2015-06-16

**Authors:** MH Aelami, M Khajehdalouee, H Yagoubian, S Amel Jamahdar, D Pittet

**Affiliations:** 1 Pediatrics, Mashhad University of Medical Sciences, Mashhad, Islamic Republic Of Iran; 2 Mashhad University of Medical Sciences, Mashhad, Islamic Republic Of Iran; 3 University of Geneva Hospitals and Faculty of Medicine, Geneva, Switzerland

## Introduction

More than two million pilgrims from different countries around the world participate in the annual Hajj in Saudi Arabia. Respiratory diseases are the most common cause of illness among pilgrims, but infection transmission can be prevented by personal hygiene.

## Objectives

To investigate the role of personal hygiene in the prevention of influenza-like illness (ILI) in Iranian pilgrims.

## Methods

In a prospective cross-sectional study conducted during the Hajj season 2012, pilgrims were randomized into two groups. The intervention group received education on personal hygiene including a hygienic package containing and alcohol-based handrub (gel or spray), surgical masks, soap, paper handkerchiefs, and user instructions; the control group did not benefit from any intervention. ILI was defined as the presence of at least two of the following during their stay: fever, cough, and sore throat. Questionnaires including demographic and clinical information were distributed among trained physicians before departure from Iran.

## Results

A total of 664 Iranian pilgrims were enrolled in the study; 306 in the intervention group and 358 in the control group. ILI was detected in 159 (52%) in the intervention group and 198 (55.3%) in the control group (p<0.001). ILI was observed less in pilgrims using a handrub in spray form (64; 41.4%) compared with those using a gel form (95; 61.2%).

## Conclusion

Hygienic education, together with the provision of a health package including surgical masks, paper handkerchiefs, soap, and a handrub can prevent ILI among pilgrims.

## Disclosure of interest

None declared.

